# Neuronal influence behind the central nervous system regulation of the immune cells

**DOI:** 10.3389/fnint.2013.00064

**Published:** 2013-09-02

**Authors:** Anahí Chavarría, Graciela Cárdenas

**Affiliations:** ^1^Laboratorio de Neuroinmunología, Departamento de Medicina Experimental, Facultad de Medicina, Universidad Nacional Autónoma de MéxicoMéxico City, México; ^2^Departamento de Neuroinfectología, Instituto Nacional de Neurología y Neurocirugía Manuel Velasco SuárezMéxico City, México

**Keywords:** neuronal immune modulation, central nervous system, neuron-microglia interaction, neuron-T cell interaction, CD200, neurotrophins, neurotransmitters, semaphorins

## Abstract

Central nervous system (CNS) has a highly specialized microenvironment, and despite being initially considered an immune privileged site, this immune status is far from absolute because it varies with age and brain topography. The brain monitors immune responses by several means that act in parallel; one pathway involves afferent nerves (vagal nerve) and the other resident cells (neurons and glia). These cell populations exert a strong role in the regulation of the immune system, favoring an immune-modulatory environment in the CNS. Neurons control glial cell and infiltrated T-cells by contact-dependent and -independent mechanisms. Contact-dependent mechanisms are provided by several membrane immune modulating molecules such as Sema-7A, CD95L, CD22, CD200, CD47, NCAM, ICAM-5, and cadherins; which can inhibit the expression of microglial inflammatory cytokines, induce apoptosis or inactivate infiltrated T-cells. On the other hand, soluble neuronal factors like Sema-3A, cytokines, neurotrophins, neuropeptides, and neurotransmitters attenuate microglial and/or T-cell activation. In this review, we focused on all known mechanism driven only by neurons in order to control the local immune cells.

## Introduction

The central nervous system (CNS) has a highly specialized immune-modulatory microenvironment, which has developed several mechanisms to protect itself from immune-mediated inflammation. This microenvironment is sustained by existing physiological and anatomical elements such as the blood-brain barrier (BBB) that limits peripheral immune cells and molecules entry; the afferent nerves of the autonomic nervous system with anti-inflammatory properties; and finally, the resident cells like astrocytes and neurons, which also contribute to the local immune privilege through the expression of anti-inflammatory suppressive factors and cell surface molecules (Carson et al., 2006).

The ability of neurons to sense changes in the brain and the body is a key factor in maintaining CNS-homeostasis. There is a large body of evidence that immune and neuronal systems communicate with each other by soluble factors as neurotransmitters, neuromodulators, and neuropeptides, or through cell-cell contact by neuroimmune regulatory molecules that can reduce or inhibit any exacerbated inflammatory response (Tian et al., 2009).

In this review, we focus on the general neuron-cell contact-dependent and contact-independent mechanisms involved in the immune modulation in order to maintain CNS immune privilege, even though microglia and astrocytes constitute the first line of defense.

## Contact-dependent mechanism for immune modulation

Neurons can display an array of membrane molecules in order to control local immune functions; these molecules can target local immune cells like microglia and astrocytes or peripheral immune cells present in the CNS. When BBB is ruptured, immune privilege is lost and neurons may come in contact with T or mononuclear cells, endangering their survival. However, neurons might modulate these immune cells by several strategies, either indirectly suppressing T-cell activation by restriction of antigen presenting properties of glial cells, directly suppressing T-cell activation, favoring a Th2 profile or promoting apoptosis of activated microglia and T-cells (Tian et al., 2009).

### Molecules inhibiting glial activation

The neuronal cell adhesion molecule (NCAM/CD56) is expressed on the surface of neurons, astrocytes and microglia (Sporns et al., 1995; Krushel et al., 1998; Chang et al., 2000a,b), and has a critical role in cell-cell adhesion, synaptic plasticity, neurite outgrowth, among other processes (Tian et al., 2009). Astrocyte-neuron interactions via NCAM lead to modulate glial scar formation by the inhibition of astrocyte proliferation *in vitro* and *in vivo* after performed stab lesions in the striatum, cerebral cortex, or hippocampus (Krushel et al., 1995, 1998). NCAM requires the activation of the glucocorticoid receptor to inhibit growth factor-induced mitogen activated protein kinase (MAPK) activity and therefore preventing astrocytic proliferation (Krushel et al., 1998). NCAM also modulates microglial activation, decreases the production of TNFα and nitric oxide (NO) after glial stimulation with lipopolysaccharide (LPS) by reducing the expression of transcription factors like c-Jun, among others (Chang et al., 2000a,b). For the mediation of glial immune responses the homophilic binding of third Ig domain of NCAM is crucial (Sporns et al., 1995; Krushel et al., 1998).

Another important molecule thought to contribute to the constitutive anti-inflammatory and regulatory environment of the brain is CD200, a highly expressed glycoprotein in the CNS, mainly in neurons (Chitnis et al., 2007; Koning et al., 2009). Neuronal CD200 down-modulates the activation state of perivascular macrophages and microglia trough the CD200 receptor (Hoek et al., 2000). Upon binding to its ligand, the tyrosine residues on the cytoplasmic tail of CD200R are phosphorylated and the downstream signaling leads to inhibition of p38 MAPK, c-Jun N-terminal kinase (JNK), and extracellular-signal-regulated kinases (ERK; Zhang et al., 2004), interfering with the activation of macrophages and microglia. Moreover, IL4 mediated neuronal CD200 expression maintains microglia in a quiescent state and anti-inflammatory/neuroprotective profile (Lyons et al., 2009). Additionally, aging leads to a depressed CD200 expression and microglial activation, favoring a pro-neurodegenerative disease environment (Cox et al., 2012). Also, defects in CD200-CD200R pathway play a critical role in neurodegenerative disease development such as multiple sclerosis (MS), Parkinson's and Alzheimer's diseases (Koning et al., 2007; Walker et al., 2009; Zhang et al., 2011).

CD22 is a regulatory sialic-acid-binding molecule that mediates neuron binding to microglia through CD45, inhibiting CD40L-induced microglial activation by suppression of the p38 and p44/42 MAPK signaling pathway and preventing microglial TNFα production after LPS stimulation (Tan et al., 2000; Mott et al., 2004; Zhu et al., 2008).

Neuronal membrane integrin-associated protein (CD47) is specially concentrated on synapses and exerts its neuroimmune functions mainly via two receptors (Tian et al., 2009). CD172 (SIRPα) ligation results in phosphatidylinositide 3-kinase (PI3K) signaling cascade activation, and reduces inflammation severity by increasing TGFβ levels, diminishing phagocytosis TNFα and INFα levels (Reinhold et al., 1995; Smith et al., 2003). Furthermore, decreased levels of CD47 are found in chronic active and inactive MS lesions, possibly favoring persistence of damage by the lack of regulation of activated microglia and macrophages (Koning et al., 2007). CD47 interaction with thrombospondin TSP, a further receptor, leads to T-cell and microglia apoptosis via CD95/CD95L pathway also reducing inflammation (Lamy et al., 2007).

Residential brain cells express CD95L (FasL) constitutively to limit possible damaging inflammatory responses. Neuronal CD95L expression induces apoptosis of infiltrating and autoreactive T-cells (Flügel et al., 2000), as well of activated microglia (Choi and Benveniste, 2004). Additionally, CD95L protects neurons from perforin-mediated T-cell cytotoxicity (Medana et al., 2001).

The expression of chemokine CX3CL1 (fractalkine) and its receptor CX3CR1 is limited to neurons and microglia, respectively (Hughes et al., 2002). CX3CL1 can be found membrane-anchored or secreted both in physiological and pathological conditions such as facial motor nerve axotomy or a toxic model of Parkinson's disease (Harrison et al., 1998; Cardona et al., 2006). CX3CL1-CX3CR1 interactions lead to the JNK MAPK pathway activation and Nrf2 recruitment suppressing the neurotoxic microglia activity and reducing neuronal death due to inflammation (Zujovic et al., 2000; Mizuno et al., 2003; Cardona et al., 2006; Noda et al., 2011).

### Molecules inhibiting immune cells

Plexin and semaphorin signaling has revealed that several members of this family are involved in immune cell processes. Among these semaphorins are Sema-3A, Sema-3E, Sema-4D, Sema-4A, Sema-6D, and Sema-7A (Roney et al., 2013). However, only Sema-3A and Sema-7A are expressed by neurons, respectively either as secreted or membrane-bound regulatory proteins that attenuate T-cell activation, proliferation, and function through T-cell receptor (TCR) signaling (Czopik et al., 2006; Lepelletier et al., 2006). Sema-3A exerts its action forming a complex with neuropilin-1 and plexin-A1 that leads to the prevention of immune response over-activation and the inhibition of human monocytes migration through the blockage of actin cytoskeleton reorganization, interfering with TCR polarization and signal transduction events by down-modulation of MAPK signaling cascades (Lepelletier et al., 2006). Also stressed neurons may induce apoptosis of INFγ or LPS activated microglia through Sema-3A secretion recruiting CD95 to lipid rafts next to neuropilin-1 (Majed et al., 2006; Moretti et al., 2008). Sema-7A, a glycosylphosphatidylinositol-linked semaphorin, negatively regulates TCR signaling and avoids activation of the ERK-MAPK pathway decreasing T-cell proliferation. Sema-7A deficient mice present T-cell hyperresponsiveness and hyperproliferation with severe experimental autoimmune encephalomyelitis pathology (Czopik et al., 2006).

Additionally, N- and E-cadherins are highly expressed in the CNS and bind to the killer cell lectin-like receptor G1 (KLRG1) on NK- and T-cells, preventing NK lysis of neurons and suppressing CD8 + T-cells antigenic proliferation and cytolytic activity (Gründemann et al., 2006; Ito et al., 2006).

Only soma and dendrites of neurons express the intercellular adhesion molecule-5 (ICAM-5/telencephalin; Tian et al., 2000). Neurons bind to T-cell through the ICAM-5-CD11a/Cd18 (LFA-1) interaction diminishing TCR dependent T-cell activation and enhancing TGFβ and INFγ expression in naïve T-cells (Tian et al., 2008). Additionally, ICAM-5 can be cleaved by activated T-cell or microglial-secreted matrix metalloproteinases-2 and -9, soluble ICAM-5 may compete with ICAM-1 costimulatory signal necessary for T-cell activation (Tian et al., 2008). Also, soluble ICAM-5 is present in blood and cerebrospinal fluid after hypoxia due to carotid artery ligation in mice and acute encephalitis in humans (Guo et al., 2000; Lindsberg et al., 2002). Moreover, ICAM-5 regulates microglia morphology and function by facilitating cell spreading and increasing CD11a/Cd18 expression (Mizuno et al., 1999).

### Neuron-mediated generation of regulatory T-cells

Regulatory T-cells (Tregs) are important in keeping CNS homeostasis in healthy and pathological conditions, and are also locally induced by glia cells and neurons (Liu et al., 2006; Saenz et al., 2010). Encephalitogenic T-cell production of IFNγ and TNFα leads to neuronal expression of TGFβ 1, CD80, and CD86, which induce encephalitogenic CD4 + T-cells to become Tregs, in a cell-to-cell dependent and antigen independent way through the TGF-β 1–TGF-β R and TCR signaling pathway (Issazadeh et al., 1998; Liu et al., 2006). Neuron-induced Tregs are able to inhibit progression of experimental autoimmune encephalomyelitis by suppression of encephalitogenic CD4 + T-cells proliferation (Liu et al., 2006).

## Contact independent mechanisms for immune modulation

Constitutive-secreted neurotrophins, neurotransmitters, and neuropeptides, as well as cytokines provide contact-independent routes for neurons to control microglial and T-cell activities.

Neurotrophins play a critical role in the control of neuronal survival, migration, and differentiation and modulate immune cell functions (Tabakman et al., 2004). Nerve growth factor (NGF), brain-derived neurotrophic factor (BDNF), and neurotrophin-3 (NT-3) can inhibit MHCII expression in microglia in hippocampal slice cultures via the low affinity p75 neurotrophin receptor (Neumann et al., 1998). NGF also down-regulates the co-stimulatory molecules CD40 and CD86 in microglia (Wei and Jonakait, 1999), is increased in cerebral spinal fluid of MS patients (Laudiero et al., 1992), and NGF treatment delays EAE onset and clinical severity (Arredondo et al., 2001). In addition, NGF arrests astrocyte cell cycle possibly restricting glial scar formation after CNS injury via the p75 neurotrophin receptor, attenuating cyclins D1 and E and preventing the degradation of cyclin-dependent kinase inhibitors p15INK and p27kip1 (Cragnolini et al., 2012). Interestingly, NT-3 has anti-inflammatory properties by diminishing microglial inducible form of NO synthase, NO, IL1-β, and TNFα levels, and phagocytic activity after LPS stimulation. NT-3 exerts its effects mainly through the TrkC receptor leading to the activation of MAPK and PI3K cascades and decreasing the NFκB-p65 activity (Tzeng and Huang, 2003; Tzeng et al., 2005).

IL10 and TGFβ cytokines have anti-inflammatory and suppressive properties that importantly regulate CNS inflammatory responses and resident cells survival (Pratt and McPherson, 1997; Strle et al., 2001). Both cytokines and their receptors are expressed by neurons and glial cells throughout the CNS (Szelényi, 2001). These regulatory molecules down-regulate microglia inhibiting the expression of MHCII, pro-inflammatory cytokines such as TNFα and IL1β, as well as NO synthesis after LPS activation (Suzumura et al., 1993; Sawada et al., 1999; Heyen et al., 2000). Furthermore, IL10 and TGFβ have an important role on Tregs and keep autoimmune T-cells under the steady state (Saenz et al., 2010).

Among the neuropeptides and neurotransmitters with modulatory properties that inhibit microglial LPS-induced pro-inflammatory factors like IL1β, IL6, TNFα, and NO, are vasoactive intestinal peptide (VIP), dopamine, norepinephrine (NE), and γ-aminobutyric acid (GABA; Färber et al., 2005; Bjurstöm et al., 2008; Delgado et al., 2008). VIP exerts its anti-inflammatory effects through the VPAC_1_ and VPAC_2_ receptors inhibiting p38 and p42/p44 MAPK and NFκB signaling cascades (Delgado et al., 2008). Also, VIP treatment avoids beta-amyloid neurodegeneration and MPTP-induced dopaminergic neuronal loss (Delgado and Ganea, 2003; Delgado et al., 2008). Additionally; VIP induces protective TH2 cells by up-regulation of macrophage B7.2 expression and Tregs in a EAE model (Delgado et al., 2004; Fernandez-Martin et al., 2006). Physiological concentrations of GABA activate functional GABA_A_ channels on encephalitogenic T-cell decreasing cell proliferation, while GABA_B_ channels activation on microglia attenuates IL6 and IL 12p40 levels after LPS stimulation (Kuhn et al., 2004; Bjurstöm et al., 2008). Functional dopamine receptors D_1_ and D_2_ are expressed by microglia and their activation lead to attenuate NO production after LPS stimulation (Kuhn et al., 2004). CNS NE levels are relevant in order to maintain tissue homeostasis since NE loss contributes to neuroinflammatory processes that lead to neurodegenerative diseases, for instance depressed mice with low NE levels respond with higher TNFα production after LPS stimulation while increasing NE levels are necessary to reduce EAE severity (Szelényi and Vizi, 2007; Simonini et al., 2010). Moreover, NE regulates microglia morphology and motility by microglial processes retraction; in this dynamic process the β 2 and α 2A receptors are involved in resting cells and activated microglia cells, respectively (Gyoneva and Traynelis, 2013).

## Conclusions

**Figure 1:**
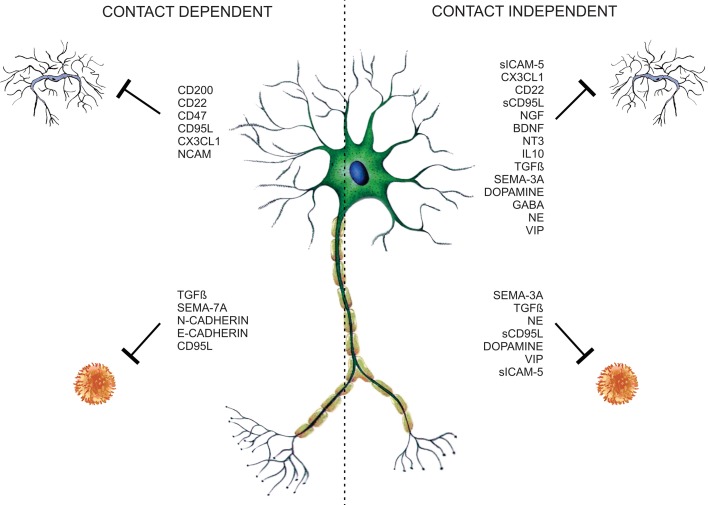
**Neuronal microglia and T-cell regulation**. Neurons control T-cell and glia functions through neuronal membrane molecules or constitutive-secreted molecules like neurotrophins, neurotransmitters, neuropeptides, semaphorins, and cytokines, constituting contact-dependent and -independent regulatory mechanisms.

Traditionally glial cells are considered to be responsible for the regulation of immune processes in the CNS. Nevertheless neurons contribute to immune modulation through contact-dependent and -independent mechanisms (Figure 1). Several neuronal secreted as well-membrane associated molecules (Table 1) are implicated in the control of glial and T-cell functions, thus contributing to CNS immune privilege.

**Table 1:** **Main neuronal immune regulatory molecules, their receptors and target cells in the CNS**.

**Neuronal molecule**	**Target cell**	**Receptor**	**References**
**CADHERIN SUPERFAMILY**			
E-cadherin	NK-cell, T-cell	KLRG1	Gründemann et al., 2006; Ito et al., 2006
N-cadherin	NK-cell, T-cell	KLRG1	Ito et al., 2006
**IMMUNOGLOBULIN SUPERFAMILY MOLECULES**			
CD22	Microglia	CD45	Mott et al., 2004
CD47	Microglia	CD172a, TSP	Smith et al., 2003; Lamy et al., 2007
CD200	Microglia	CD200R	Hoek et al., 2000; Rijkers et al., 2008
ICAM-5	T-cell	CD11a/Cd18	Mizuno et al., 1999; Tian et al., 2000, 2008
NCAM	Microglia, Astrocyte	NCAM	Sporns et al., 1995; Krushel et al., 1998; Chang et al., 2000a
**TUMOR NECROSIS FACTOR FAMILY**			
CD95L	Microglia, T-cell	CD95	Choi and Benveniste, 2004
**CYTOKINES AND CHEMOKINES**			
IL10	Microglia, T-cell	IL10R	Strle et al., 2001
TGFβ	Microglia, T-cell	TGFβ R	Pratt and McPherson, 1997; Liu et al., 2006
CX3CL1	Microglia	CX3CR1	Hughes et al., 2002
**NEUROTRANSMITTERS AND NEUROPEPTIDES**			
GABA	Microglia	GABA_A_, GABA_B_	Färber and Kettenmann, 2005
Dopamine	Microglia, T-cell	D_1_, D_2_, D_3_, D_4_, D_5_	Färber et al., 2005
NE	Microglia, Astrocyte, T-cell	α_1A_, α_2A_, β_1_, β_2_	Färber et al., 2005; Gyoneva and Traynelis, 2013
VIP	Astrocyte, T-cell	VPAC_1_, VPAC_2_	Delgado et al., 2004, 2008
**NEUROTROPHINS**			
NGF	Microglia, Astrocyte	p75, NTR, TrkA	Neumann et al., 1998; Althaus and Richter-Landsberg, 2000; Cragnolini et al., 2012
BDNF	Microglia, Astrocyte	p75, NTR, TrkB	Neumann et al., 1998; Althaus and Richter-Landsberg, 2000
NT-3	Microglia	p75, NTR, TrkB, TrkC	Neumann et al., 1998; Althaus and Richter-Landsberg, 2000; Tzeng and Huang, 2003
**SEMAPHORINS**			
Sema-3A	Microglia, T-cell	Neuropilin-1 and plexin-A1	Lepelletier et al., 2006
Sema-7A	T-cell	Plexin-C1, α 1β 1 integrin	Czopik et al., 2006

BDNF, brain-derived neurotrophic factor; ICAM-5, intercellular adhesion molecule-5; GABA, γ-aminobutyric acid; NCAM, neuronal cell adhesion molecule; NE, norepinephrine; NGF, nerve growth factor; NT-3, neurotrophin-3; Sema-3A, semaphorin-3A; Sema-7A, semaphorin-7A; VIP, vasoactive intestinal peptide.

### Conflict of interest statement

The authors declare that the research was conducted in the absence of any commercial or financial relationships that could be construed as a potential conflict of interest.
